# Sintering Mechanism, Microstructure Evolution, and Mechanical Properties of Ti-Added Mo_2_FeB_2_-Based Cermets

**DOI:** 10.3390/ma13081889

**Published:** 2020-04-17

**Authors:** Yupeng Shen, Zhifu Huang, Lei Zhang, Kemin Li, Zhen Cao, Peng Xiao, Yongxin Jian

**Affiliations:** State Key Laboratory for Mechanical Behavior of Materials, Xi’an Jiaotong University, Xi’an 710049, China; ilovehit3@126.com (Y.S.); xjtulei@outlook.com (L.Z.); kmin2015@stu.xjtu.edu.cn (K.L.); zcao_xjtu@163.com (Z.C.); xiaopeng_0207@163.com (P.X.)

**Keywords:** Mo_2_FeB_2_-based cermets, Ti addition, sintering mechanism, microstructure, mechanical properties

## Abstract

Four series of Mo_2_FeB_2_-based cermets with Ti contents between 0 wt.% and 1.5 wt.% in 0.5 wt.% increments were prepared by in situ reaction and liquid phase sintering technology. Influences of Ti on microstructure and mechanical properties of cermets were studied. It was found that Ti addition increases formation temperatures of liquid phases in liquid-phase stage. Ti atoms replace a fraction of Mo atoms in Mo_2_FeB_2_ and the solution of Ti atoms causes the Mo_2_FeB_2_ crystal to be equiaxed. In addition, the cermets with 1.0 wt.% Ti content exhibit the smallest particle size. The solution of Ti atoms in Mo_2_FeB_2_ promotes the transformation of Mo_2_FeB_2_ particles from elongated shape to equiaxed shape. With Ti content increasing from 0 wt.% to 1.5 wt.%, the hardness and transverse rupture strength (TRS) first increase and then decrease. The maximum hardness and TRS occur with 1.0 wt.% Ti content. However, the fracture toughness decreases as Ti content increases. The cermets with 1.0 wt.% Ti content show excellent comprehensive mechanical properties, and the hardness, fracture toughness, and TRS are HRA 89.5, 12.9 MPa∙m^1/2^, and 1612.6 MPa, respectively.

## 1. Introduction

Borides possess high hardness and stable chemical properties. Thus, borides are a potential wear-resistant material and have been studied by many scholars [1,2,3]. Nevertheless, borides have intrinsic brittleness and poor sinterability. It is difficult to fabricate boride-based cermets due to these defects [4]. Reaction boronizing sintering is an efficient method to generate ternary borides by the reaction of binary borides and metals in the sintering process [5]. Ternary boride-based cermets exhibit low density, high fracture toughness, high hardness, excellent wear resistance and good corrosion resistance [6,7,8,9,10]. Therein, Mo_2_FeB_2_-based cermets have attracted relatively more attention for their low cost, little abrasion value, and excellent mechanical properties [4,11,12,13]. Mo_2_FeB_2_-based cermets are widely used in can tools and copper extrusion molds. Thus, Mo_2_FeB_2_-based cermets are widely studied [14,15,16,17,18].

Mo_2_FeB_2_-based cermets are composed with two phases: Mo_2_FeB_2_ and Fe. Thus, Mo_2_FeB_2_-based cermets have the advantages of metal and ceramic [19]. Alloying is a common method to increase the mechanical properties of cermets. At present, V, Cr and Mn are the most common doped elements. It was found that V atoms replace partial Mo atoms in Mo_2_FeB_2_, and decrease particle size of Mo_2_FeB_2_ [20]. The previous work revealed that Mn atoms replace a fraction of Mo atoms in Mo_2_FeB_2_ particles, and the addition of Mn increases the wettability of liquid phase on Mo_2_FeB_2_ particles [21,22,23]. It was reported that Cr atoms replace a fraction of Mo atoms in Mo_2_FeB_2_ particles, and the addition of Cr decreases the particle size and increases the sphericity of Mo_2_FeB_2_ hard phase [24,25]. Through analyses, a general rule can be obtained that doped elements replace partial Mo atoms in Mo_2_FeB_2_ hard phase, since the atomic radii of V (1.92 Å), Mn (1.79 Å), and Cr (1.85 Å) are close to the atomic radius of Mo (2.01 Å) [26]. It is natural to assume that partial Mo atoms in Mo_2_FeB_2_ hard phase can be replaced by other atoms which have similar atomic radii. According to periodic table, the atomic radius of Ti is 2.00 Å and it is very close to the atomic radius of Mo. Thus, Ti may be a new doped element which can replace partial Mo atoms. The Mo_2_FeB_2_ hard phase shows a structure of U_3_Si_2_ and belongs to a tetragonal system [27,28,29]. Considering the strong bonding existing within Mo_2_FeB_2_ that contributes to its incredible properties [28,29,30,31], the substitution of atoms may have an important effect on the performance of cermets. To date, however, adding Ti in Mo_2_FeB_2_-based cermets is barely reported.

Herein, four series of cermet samples with xTi (x = 0, 0.5, 1.0, 1.5, wt.%) contents were prepared by in situ reaction and liquid phase sintering technology. Influences of Ti on microstructure and mechanical properties of cermets are studied in detail.

## 2. Materials and Experimental Procedure

### 2.1. Materials

**Figure 1:**
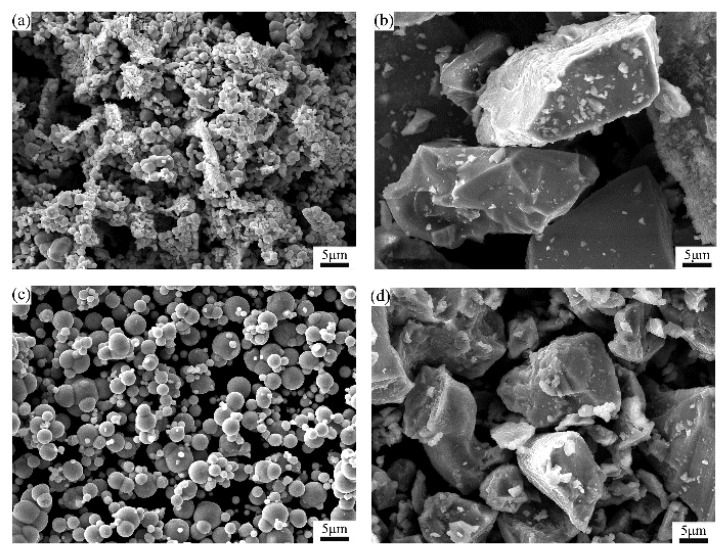
Scanning electron microscopy (SEM) micrographs of raw powders: (**a**) Mo; (**b**) FeB; (**c**) Fe; (**d**) Ti.

In this paper, Mo, FeB, Fe and Ti powders, which are available on the market, were used as raw materials. Figure 1 shows the micromorphologies of the raw powders. It can be seen that the shape of Fe and Mo powders are spherical. However, the shape of Ti and FeB powders are irregular. Table 1 shows characteristics of the raw materials. The nominal compositions used in this work was Fe_46.5−x_ Mo_47.5_B_6_Ti_x_ (x = 0, 0.5, 1.0, 1.5, wt.%).

### 2.2. Fabrication Process

Raw powders, which were weighed according to above proportion, were put into a planetary ball mill for grinding. After that, the grated powders were dried using a rotary evaporator. A semi-automatic press was used to press the powders into green compacts. Afterwards, the samples were placed in vacuum sintering furnace for sintering. Figure S1 shows the sintering process curve (Supplementary Materials).

### 2.3. Characterization

The phase-transition temperatures of samples were determined by differential scanning calorimetry (DSC, STA 449F5, NETZSCH, Bavaria, Germany). The samples were heated from room temperature to 1300 °C. The phase analyses were performed via X-ray diffraction analysis (XRD, D8 Advance, Bruker, Billerica, MA, USA) using Cu Kα radiation at 40 kV as an X-ray source. Scanning electron microscopy (SEM, SU3500, Hitachi, Tokyo, Japan) was used to observe the microstructure of Mo_2_FeB_2_-based cermets and bending fracture surface morphology. The particle size and sphericity of Mo_2_FeB_2_ hard phase were measured via Image analysis software (Image Pro Plus, Version 6.0, Media Cybernetics, Rockville, MD, USA) [32,33,34]. The measurement methods and calculation formulas are provided in supplementary information. ImageJ software was utilized to calculate the volume fraction of Mo_2_FeB_2_ particles and 20 SEM micrographs were used for each group of specimens to obtain statistical results. The relative densities of cermets were determined as per Archimedes technique [35]. The calculation formula is provided in supplementary information. The distribution of various elements in cermets was determined by electron probe microanalysis (EPMA, JXA-8230, JEOL, Tokyo, Japan).

The hardness of Mo_2_FeB_2_-based cermets was measured under a hardness tester (MX1000, Jinan, China), and each sample was measured at least 5 times. Fracture toughness (K_IC_) and transverse rupture strength (TRS) tests of Mo_2_FeB_2_-based cermets at room temperature were conducted using a three-point bend test [35], whose measurement methods and calculation formulas are provided in supplementary information.

## 3. Results and Discussion

### 3.1. Sintering Mechanism

**Figure 2:**
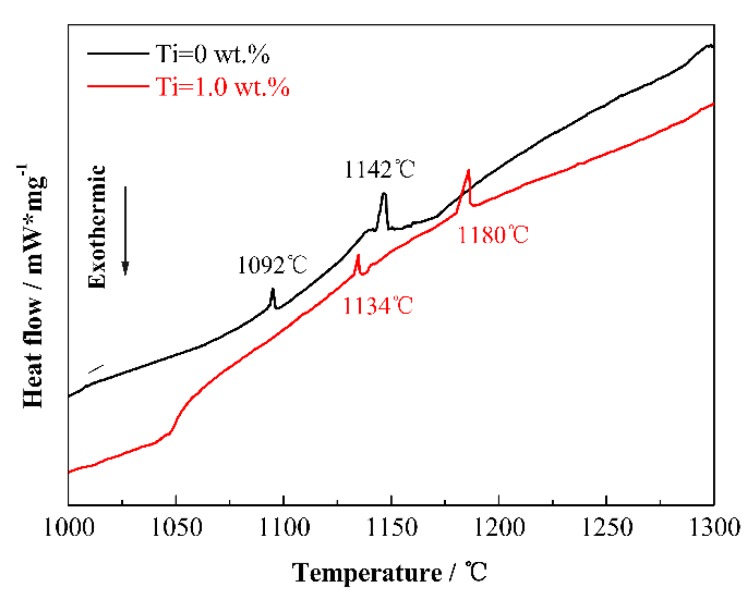
DSC curves of powders with 0 and 1.0 wt.% Ti contents.

DSC curves of powders with 0 wt.% and 1.0 wt.% Ti contents are presented in Figure 2. It can be seen that from Figure 2, the trends of the two curves are basically the same, and both of the thermal curves have two endothermic peaks. However, the difference is that the temperatures of forming liquid phases are changed. The endothermic peaks of milled powders with 1.0 wt.% Ti content occur at higher temperature compared to that without Ti. With the sintering temperature increasing, L_1_ and L_2_ occur as follows [36].

(1) $$\gamma –\mathrm{Fe}+{\mathrm{Fe}}_{2}B\to L_{1}$$

(2) $$\gamma –\mathrm{Fe}+\;L_{1}+\;{\mathrm{Mo}}_{2}{\mathrm{FeB}}_{2}\to L_{2}+{\mathrm{Mo}}_{2}{\mathrm{FeB}}_{2}$$

It can be speculated from Figure 2 that Ti addition increases reaction temperatures of liquid phases. It was reported that the solid solubility of Ti in γ–Fe is very low [37,38], and it is not conducive to the inter-diffusion among elements [39]. Thus, Ti addition can inhibit the inter-diffusion in the sintering, as a result, the liquid-phase reactions take place at a higher temperature. It is worth noting that the relative density and particle size of cermets are affected by two liquid-phase reactions. As per the literature [16,40,41], grain rearrangement of Mo_2_FeB_2_ particles occurs in L_1_ such that the densification of Mo_2_FeB_2_-based cermets increases rapidly. On the other hand, the growth of Mo_2_FeB_2_ particles is mainly achieved via dissolution and precipitation of Mo_2_FeB_2_ grains in L_2_. As a result, the increase of reaction temperature of L_1_ is not conducive to the densification of cermets with Ti addition. However, the increase of forming temperature of L_2_ contributes to grain refinement of cermets. The influence mechanism of Ti will be systematically described below.

### 3.2. Microstructure Evolution

**Figure 3:**
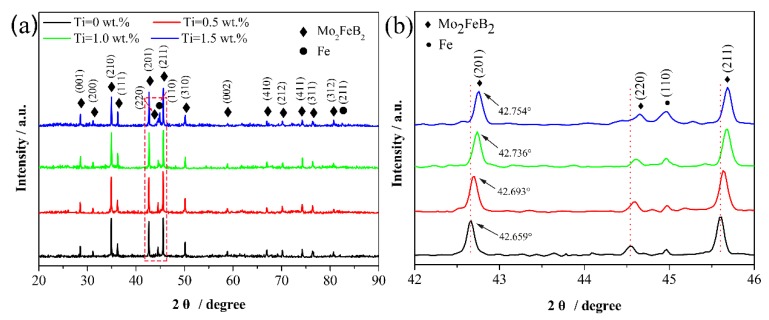
XRD patterns of Mo_2_FeB_2_-based cermets with various Ti contents: (**a**) integral XRD patterns; (**b**) Local XRD patterns range from 42° to 46°.

Figure 3 shows XRD patterns of Mo_2_FeB_2_-based cermets with various Ti contents. It can be seen that from Figure 3a, cermets with various Ti contents have the same phase compositions. The main phases are Mo_2_FeB_2_ (JCPDS 89-3630) and Fe (JCPDS 87-0722). Figure 3b shows the local XRD curves with diffraction angle between 42° and 46°. It can be seen that from Figure 3b, with the increase of Ti content, the diffraction angles of Mo_2_FeB_2_ gradually increase. For instance, the diffraction angle of main peak (201) of Mo_2_FeB_2_ hard phase without Ti is 42.659° while that with 1.5 wt.% content Ti is 42.754°. The increase of diffraction angle means the shrinkage of the lattice of Mo_2_FeB_2_. The shrinkage of the lattice is caused by the substitution of atoms (Ti) with smaller radii for atoms (Mo) with larger radius, which is consistent with what is envisaged in the experiment.

**Figure 4:**
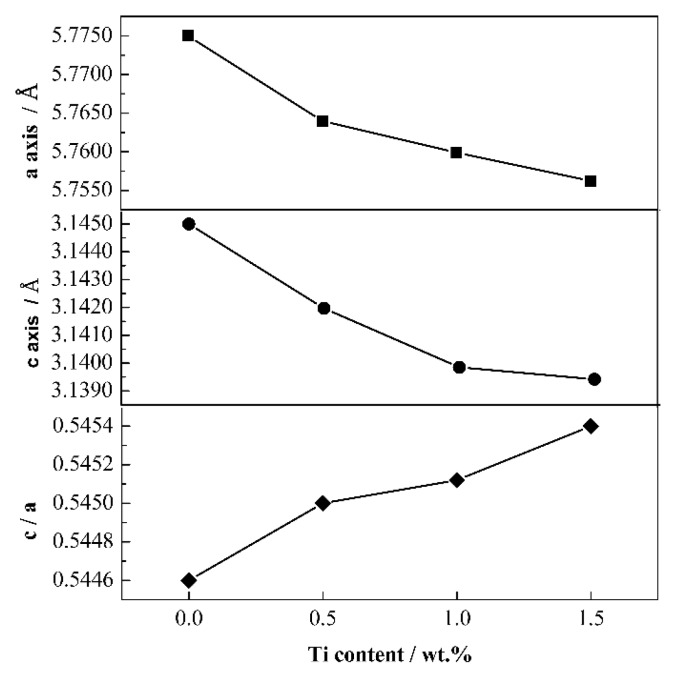
Lattice constants of Mo_2_FeB_2_ crystal with different Ti contents.

Figure 4 shows the lattice constants of Mo_2_FeB_2_ crystal with different Ti contents. As shown, the lattice constant a and c of Mo_2_FeB_2_ crystal both decrease with increasing Ti content. The lattice constant a of Mo_2_FeB_2_ decreases from 5.7750 Å to 5.7562 Å and the lattice constant c decreases from 3.1450 Å to 3.1394 Å, which is consistent with diffraction peaks shift of Mo_2_FeB_2_ hard phase shown in Figure 3. However, the c/a ratio gradually increases from 0.5446 to 0.5454. The c/a approaching 1 indicates that the crystal has become equiaxed. Thus, the solution of Ti into Mo_2_FeB_2_-based cermets causes Mo_2_FeB_2_ crystal to be equiaxed.

**Figure 5:**
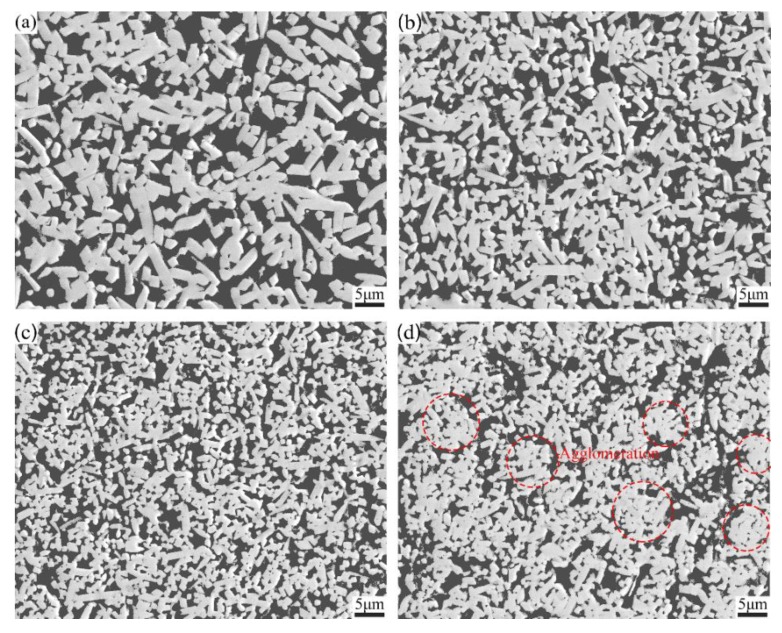
Microstructure of Mo_2_FeB_2_-based cermets with various Ti contents: (**a**) 0 wt.%; (**b**) 0.5 wt.%; (**c**) 1.0 wt.%; (**d**) 1.5 wt.%.

Figure 5 shows the microstructure of cermets with various Ti contents. All Mo_2_FeB_2_-based cermets are composed with two phases: Mo_2_FeB_2_ and Fe. The results are consistent with XRD analyses shown in Figure 3. For cermets with Ti content between 0 wt.% and 1.0 wt.%, Mo_2_FeB_2_ particles are equally distributed in the cermets. Nevertheless, aggregation of Mo_2_FeB_2_ hard phases occurs when Ti content reaches 1.5 wt.%.

**Figure 6:**
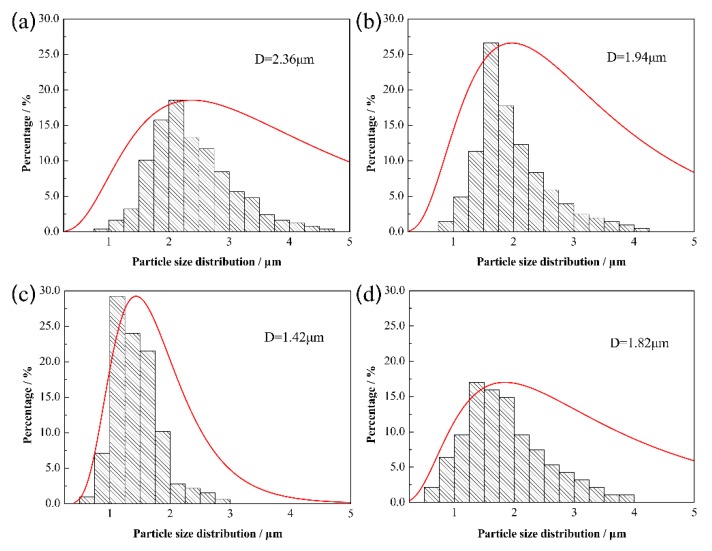
Particle size distribution of Mo_2_FeB_2_ hard phase with different Ti contents: (**a**) 0 wt.%; (**b**) 0.5 wt.%; (**c**) 1.0 wt.%; (**d**) 1.5 wt.%.

Moreover, the particle size of the Mo_2_FeB_2_ hard phase varies with Ti addition. With the increase of Ti content, the particle size of Mo_2_FeB_2_ hard phase first decreases and then increases. The cermets exhibit the smallest particle size when Ti content is 1.0 wt.%. In order to further analyze the variation trend of particle size of the Mo_2_FeB_2_ hard phase, the statistical results of particle size distribution of Mo_2_FeB_2_ are presented in Figure 6 and will be discussed in detail below. As shown in Figure 6, with the increase of Ti content from 0 wt.% to 1.0 wt.%, the range of particle size distribution becomes narrower and then becomes wider at Ti content of 1.5 wt.%. In addition, the average particle size of Mo_2_FeB_2_-based cermets first decreases and then increases. The minimum average particle size of 1.42 μm is obtained when Ti content is 1.0 wt.%. The causes of Mo_2_FeB_2_ particle size variation are complex. When Ti content is between 0 wt.% and 1.0 wt.%, the amount of liquid phase is enough in high-temperature phase. The growth mechanism of Mo_2_FeB_2_ particles is solution–precipitation [36]. Thus, the particle size of Mo_2_FeB_2_ particles depends on the solution-precipitation reaction. As a result of the addition of Ti, the formation temperature of L_2_ increases, which is harmful to the growth of Mo_2_FeB_2_ particles. Therefore, the particle size of Mo_2_FeB_2_ particle decreases. When Ti content is 1.5 wt.%, the amount of liquid phase decreases further. The distance among Mo_2_FeB_2_ grains becomes smaller so as to Mo_2_FeB_2_ grains tend to merge and grow [4,17], and the Mo_2_FeB_2_ grains no longer grow through the solution–precipitation mechanism. Thus, the Mo_2_FeB_2_ will be coarser as particles aggregate.

**Figure 7:**
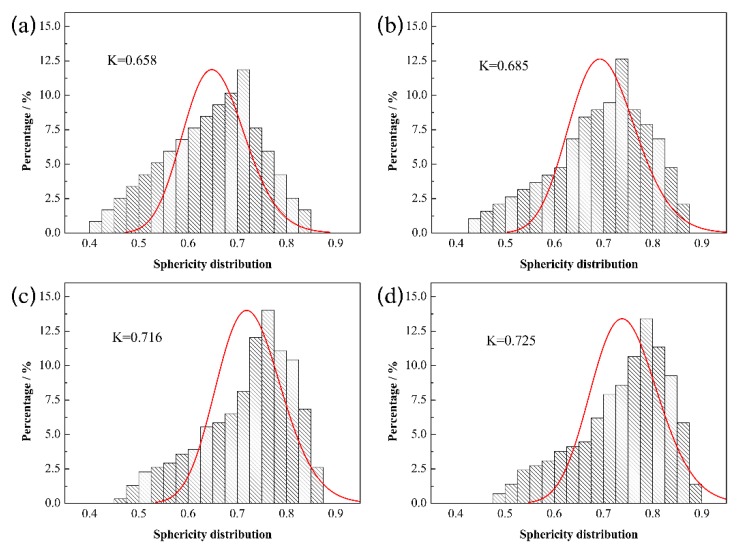
Sphericity distribution of Mo_2_FeB_2_ hard phase with different Ti contents: (**a**) 0 wt.%; (**b**) 0.5 wt.%; (**c**) 1.0 wt.%; (**d**) 1.5 wt.%.

Figure 7 shows the sphericity distribution of Mo_2_FeB_2_ hard phase with various Ti contents. It can be seen that from Figure 7, the values of lower and upper limits of sphericity distribution range gradually increase with Ti content increasing, while the range of sphericity distribution becomes narrower. And the average sphericity increases from 0.658 to 0.725. The closer the sphericity is to 1, the more equiaxed the Mo_2_FeB_2_ particles are, indicating that the shape of Mo_2_FeB_2_ particles gradually becomes equiaxed with Ti content increasing. It was reported that the morphologies of particles are influenced by their surface energies [42,43]. Thus, due to the addition of Ti, the surface energies of Mo_2_FeB_2_ particles may be changed. Ti addition reduces the preferred orientation of Mo_2_FeB_2_ particles. Takagi and Yu [15,20] reported that the shape of Mo_2_FeB_2_ particles depends on the degree of grain orientation. Thereby, as per the calculation results for lattice constant of Mo_2_FeB_2_ crystal shown in Figure 4, the phenomenon of equiaxed particles can be reasonably explained.

**Figure 8:**
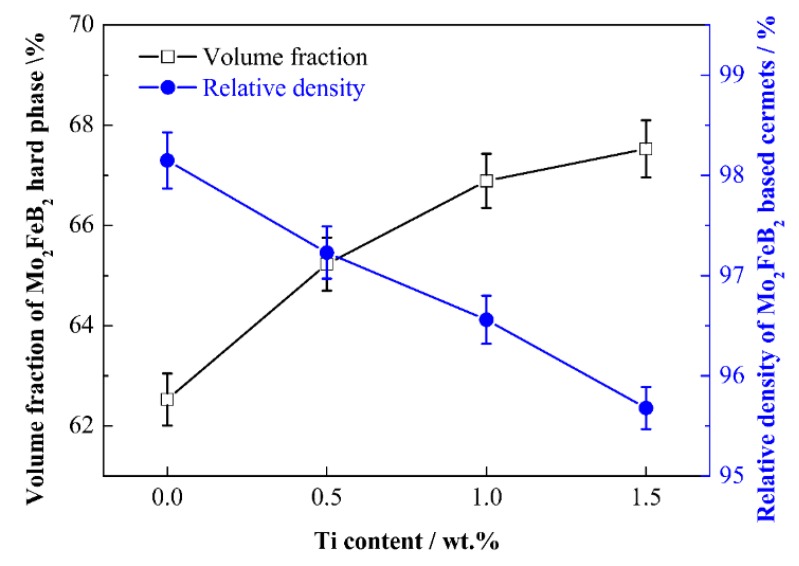
Volume fraction of Mo_2_FeB_2_ hard phase and relative density of Mo_2_FeB_2_-based cermets with various Ti contents.

Figure 8 shows the volume fraction of Mo_2_FeB_2_ particles and relative density of Mo_2_FeB_2_-based cermets with various Ti contents. As Ti content increases from 0 wt.% to 1.5 wt.%, the volume fraction of Mo_2_FeB_2_ hard phase increases. The reason is that Fe content decreases with Ti content increasing, resulting in the increase of the content of Mo_2_FeB_2_. Nevertheless, it can also be seen that the relative density of cermets shows the opposite trend. The relative density keeps falling with Ti content increasing. There are two reasons for the drop in relative density. The first reason is that the increase of Ti content leads to the decrease of liquid phase in cermets. The lesser liquid phase does not flow well enough to fill the holes efficiently. The second reason is that the increase of formation temperature of L_1_ is not conducive to the densification of cermets due to Ti addition. Thus, the relative density of cermets decreases.

**Figure 9:**
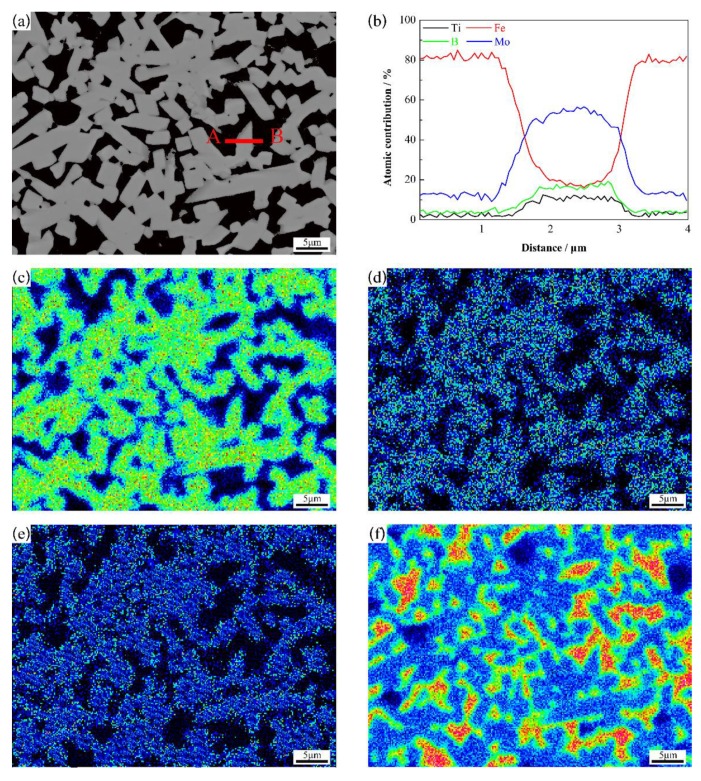
Electron probe microanalysis (EPMA) analyses of Mo_2_FeB_2_-based cermets with 1.0 wt.% Ti content: (**a**) SEM picture; (**b**) line scan analyses along line segment AB of (**a**); (**c**–**f**) mapping of Mo, B, Ti and Fe element of (**a**), respectively.

EPMA analyses of Mo_2_FeB_2_-based cermets with 1.0 wt.% Ti content are presented in Figure 9. As shown, the distribution of Mo, B and Ti are mainly the same. However, Fe has the opposite trend. It can be concluded that, Ti, Mo, and B are mainly distributed in the Mo_2_FeB_2_ hard phase while Fe is mainly distributed in the binder phase.

The chemical compositions of the Mo_2_FeB_2_ hard phase with different Ti contents are measured by EPMA, as shown in Table 2. Fe, Ti and B content in Mo_2_FeB_2_ all increase as Ti content increases, while the Mo content decreases. Therefore, Ti atoms are supposed to replace partial Mo atoms in Mo_2_FeB_2_. The results are consistent with the analyses above.

### 3.3. Mechanical Properties

**Figure 10:**
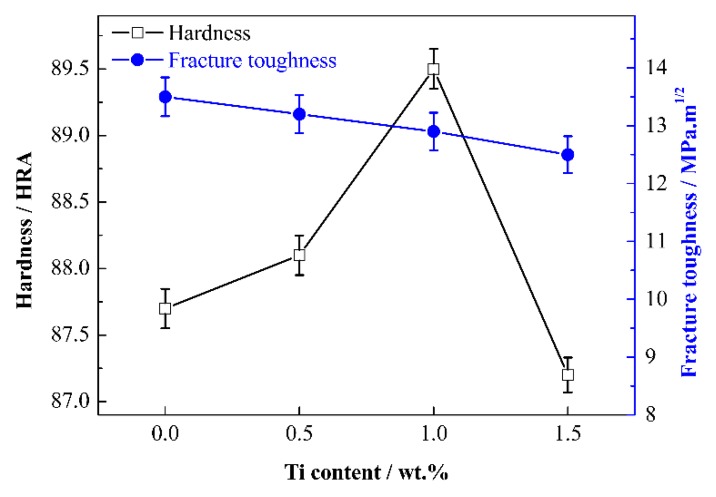
Hardness and fracture toughness of Mo_2_FeB_2_-based cermets with various Ti contents.

Hardness and fracture toughness of cermets with various Ti contents are shown in Figure 10. As shown, with the increase of Ti content, the hardness of Mo_2_FeB_2_-based cermets first increases and then decreases. The maximum hardness of 89.5 HRA is obtained when Ti content is 1.0 wt.%. As is well known, high volume fraction of hard phase, small particle size, and high relative density in cermets are beneficial to the improvement of hardness. In this work, with Ti content increasing from 0 wt.% to 1.0 wt.%, the relative amount of Mo_2_FeB_2_ hard phase increases and the particle size of Mo_2_FeB_2_ particles decreases, resulting in positive effects on the improvement of hardness. However, the drop of relative density is harmful to hardness. Therefore, the hardness of Mo_2_FeB_2_-based cermets increases with the increase of Ti content. When Ti content is 1.5 wt.%, the increase of relative amount of Mo_2_FeB_2_ hard phase is conducive to the improvement of hardness, while particle size of Mo_2_FeB_2_ particles increases and relative density decreases further. Adverse effects predominate in the increase of hardness. Thus, the hardness of Mo_2_FeB_2_-based cermets is greatly reduced when Ti content is 1.5 wt.%.

With the increase of Ti content from 0 wt.% to 1.5 wt.%, the fracture toughness of Mo_2_FeB_2_-based cermets keeps going down, while the decline scope is small. Because Mo_2_FeB_2_ hard phase is brittle, the fracture toughness of Mo_2_FeB_2_-based cermets is mainly provided by Fe binder phase. Thus, the high content of Fe binder phase is beneficial to the increase of fracture toughness of cermets. The fracture toughness of cermets is also affected by factors like relative density of cermets and shape of Mo_2_FeB_2_ particles. Both the high relative density of Mo_2_FeB_2_-based cermets and equiaxed Mo_2_FeB_2_ particles are beneficial to the increase of fracture toughness of cermets. The relative amount of Fe binder phase and relative density of cermets both decrease with Ti content increasing, which is harmful to fracture toughness. The factor favorable to the improvement of fracture toughness is that Mo_2_FeB_2_ particles become equiaxed. On the whole, the negative effects predominate with the increase of fracture toughness. Thus, the fracture toughness decreases with increasing Ti content.

**Figure 11:**
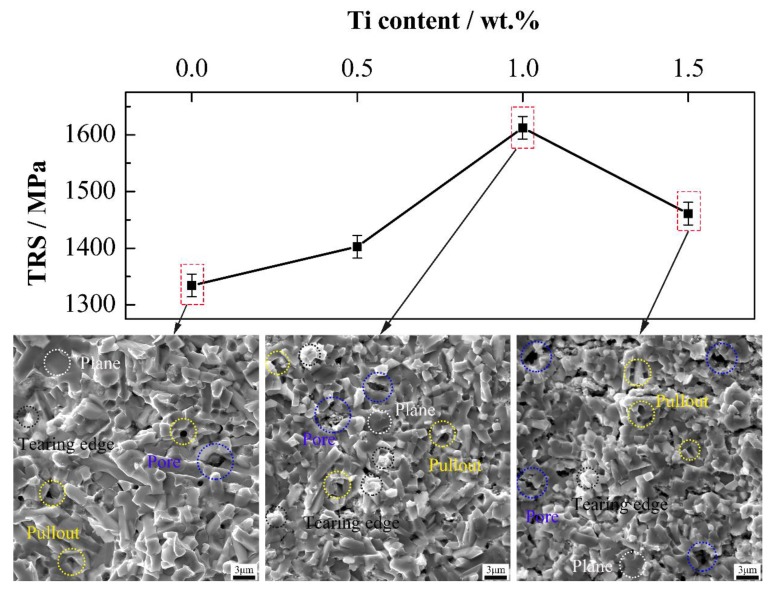
TRS of Mo_2_FeB_2_-based cermets and corresponding fractograph after three-point bending tests with different Ti contents.

Figure 11 displays the TRS of cermets with various Ti contents and the corresponding fractograph after three-point bending tests. In the bending fracture surface morphology images, the tearing edge, pullout, transgranular fracture, and pore are marked with dotted circles in different colors. This line chart shows that with the increase of Ti content from 0 wt.% to 1.5 wt.%, the TRS first increases and then decreases. The maximum TRS of 1612.6 MPa occurs at 1.0 wt.% Ti content. As Ti content is between 0 wt.% and 1.0 wt.%, fine grained, equably distributed, and equiaxed Mo_2_FeB_2_ particles are beneficial to the improvement of TRS. The uniform distribution of Mo_2_FeB_2_ particles makes the stress scatter in more particles under bend deformation, resulting in more uniform deformation [44,45]. Meanwhile, the equiaxed Mo_2_FeB_2_ particles also reduce the stress concentration. Furthermore, due to the equiaxed Mo_2_FeB_2_ particles, the microcracks are more likely to propagate in the Fe binder phase, which absorbs a lot of energy and leads to the improvement of TRS of Mo_2_FeB_2_-based cermets. Additionally, presence of equiaxed Mo_2_FeB_2_ hard phase in cermets will change the direction of crack propagation, causing the crack bridging, branching and deflection [46,47]. As shown in the fracture surface with 0 wt.% and 1.0 wt.% Ti contents, less pores are observed in cermets without Ti, which shows good densification of cermets. More tearing edges generated by the plastic deformation of Fe, are observed in cermets with 1.0 wt.% Ti content, indicating that cermets consume more energy in the process of fracture. Besides, more transgranular fracture is observed, which shows a firm interfacial binding and an increase of TRS. When Ti content is 1.5 wt.%, the decrease of the relative density of cermets and agglomeration growth of Mo_2_FeB_2_ hard phase cause the big drop in TRS. The low relative density of cermets can increase the amount of pores, resulting in a stress concentration. The large size of Mo_2_FeB_2_ grains caused by grain agglomeration is not conducive to the TRS of cermets as per the Hall–Petch theory. As shown in fractograph of 1.5 wt.% Ti content, more pores are seen in cermets, indicating more stress concentration and are more easily ruptured. In addition, more pullout, which is determined by interfacial bonding strength between Mo_2_FeB_2_ and Fe, is also observed in cermets. These results show that the wettability of Fe on Mo_2_FeB_2_ particles is poor, which is not conducive to TRS improvement.

## 4. Conclusions

(1) The addition of Ti increases temperatures of forming liquid phases in the sintering process.

(2) Ti elements are mainly distributed in Mo_2_FeB_2_ and Ti atoms replace a fraction of Mo atoms in Mo_2_FeB_2_. The solution of Ti atoms causes the Mo_2_FeB_2_ crystal to be equiaxed. When Ti content is 1.0 wt.%, the cermets exhibit the smallest particle size. Moreover, the solution of Ti atoms in Mo_2_FeB_2_ promotes the transformation of Mo_2_FeB_2_ particles from elongated shape to equiaxed shape.

(3) With Ti content increasing from 0 wt.% to 1.5 wt.%, the hardness and TRS first increase and then decrease. The maximum hardness and TRS occur with 1.0 wt.% Ti content. However, the fracture toughness decreases with Ti content increasing. The cermets with 1.0 wt.% Ti content show the excellent comprehensive mechanical properties, and the hardness, fracture toughness, and TRS are HRA 89.5, 12.9 MPa∙m^1/2^, and 1612.6 MPa, respectively.

## Figures and Tables

**Table 1:** Characteristics of raw materials.

Powder	Average Particle Size (μm)	Chemical Composition (wt.%)	Manufacturer
Mo	2	Mo ≥ 99.95, Fe < 0.005, Si < 0.002	Changsha Tianjiu Metal Material Corp., Ltd. Changsha, China
Ti	24	Ti ≥ 99.50, O ≤ 0.25, Si ≤ 0.02	
Fe	5	Fe ≥ 99.81, C < 0.015, O < 0.16	
FeB	45	B = 20.05, Si < 0.36, C < 0.36	

**Table 2:** Chemical compositions of Mo_2_FeB_2_ hard phase with different Ti contents (wt.%).

Ti Content	Ti	Fe	Mo	B
0	0	20.74	71.11	8.15
0.5	0.72	20.94	70.13	8.21
1.0	1.38	21.57	68.78	8.27
1.5	1.89	21.87	67.91	8.33

## Supplementary Materials

The following are available online at https://www.mdpi.com/1996-1944/13/8/1889/s1, Figure S1: Sintering curve of Mo_2_FeB_2_-based cermets.
