# Supplementary material for: Sintering Mechanism, Microstructure Evolution, and Mechanical Properties of Ti-Added Mo2FeB2-Based Cermets
Source: Materials (Basel). 2020 Apr 17;13(8):1889. doi: 10.3390/ma13081889 (PMC7215874; doi:10.3390/ma13081889)
Supplement: Supplementary file 1 [file materials-13-01889-s001.pdf]

materials

Supplementary Material

Sintering Mechanism, Microstructure Evolution, and Mechanical Properties of Ti-Added Mo_2FeB_2-Based Cermets

Yupeng Shen, Zhifu Huang *, Lei Zhang, Kemin Li, Zhen Cao, Peng Xiao and Yongxin Jian *

State Key Laboratory for Mechanical Behavior of Materials, Xi'an Jiaotong University, Xi'an 710049, China; ilovehit3@126.com (Y.S.); xjtulei@outlook.com (L.Z.); kmin2015@stu.xjtu.edu.cn (K.L.); zcao_xjtu@163.com (Z.C.); xiaopeng_0207@163.com (P.X.)

* Correspondence: zhifuhuangxjtu@163.com (Z.H.); yxjian@xjtu.edu.cn (Y.J.)

Received: 10 March 2020; Accepted: 14 April 2020; Published: date

1. Experimental procedure:

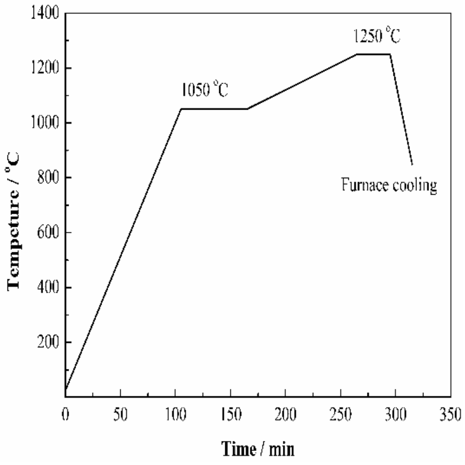

Figure S1. Sintering curve of Mo_2FeB_2-based cermets.

2. Characterization

The particle size of Mo_2FeB_2 hard phase was measured using the equivalent circle method. The equivalent circle method states that elongated particles are equivalent to the circular particles with the same area, and that the diameter of the equivalent circle is the particle size of Mo_2FeB_2 hard phase. The particle size was calculated using Equation (1):

$$D = 2\sqrt{\frac{S}{\pi}} \quad (1)$$

where S is the area of a single Mo_2FeB_2 hard phase.

The sphericity (K) was calculated using Equation (2):

$$K = \frac{4\pi S}{L^2} \quad (2)$$

where S and L are the area and perimeter of single Mo_2FeB_2 hard phase, respectively. The closer the value is to 1, the closer the shape of Mo_2FeB_2 hard phase is to a circle. More than 500 grains were measured for each sample to obtain the statistical results.

The relative density of Mo_2FeB_2-based cermets was measured according to Archimedes technique. The relative density (ρ) was calculated using Equation (3):

$$\rho = \frac{m_1 - m_2}{m_3 - m_2} \tag{3}$$

where m_1, m_2 and m_3 are dry weight, suspending weight, and wet weight of the samples, respectively. The weight of samples was measured using an electronic analytical balance (XS105, Mettler Toledo, Zurich, Switzerland) with the accuracy of 0.01 mg.

The hardness of Mo_2FeB_2-based cermets was measured under a hardness tester (MX1000, Jinan, China), and each sample was measured at least 5 times. Fracture toughness (K_{IC}) at room temperature was obtained using Single Edge Notched Beam (SENB) method with line incision. Five specimens for each kind of cermets, with dimensions of 2.5 mm × 5 mm × 25 mm, were prepared and tested to ensure the reproducibility of data. The measurements were performed using a three-point bend test with a cross-head speed of 0.1 mm/min (INSTRON-1195, Boston, MA, USA). Fracture toughness (K_{IC}) was calculated using Equations (4) and (5):

$$K_{IC} = Y\frac{3Fl}{2bh^2}a^{1/2} \tag{4}$$

$$Y = 1.93 - 3.07\frac{a}{h} + 14.53(\frac{a}{h})^2 - 25.11\left(\frac{a}{h}\right)^3 + 25.80(\frac{a}{h})^4 \tag{5}$$

where Y is the geometrical factor related to the crack depth ratio, F is the critical rupture load, b, l and h are width, length and thickness of bar, respectively, and a is the initial crack length.

The transverse rupture strength (TRS) tests of Mo_2FeB_2-based cermets at room temperature were conducted using a three-point bend test with a cross-head speed of 0.1 mm/min. Five specimens, each with dimensions of 3 mm × 4 mm × 22 mm, were prepared and tested to ensure the reproducibility of data. The TRS (δ) was calculated using Equation (6):

$$\delta = \frac{3Fl}{2bh^2} \tag{6}$$

where F is the critical rupture load, l, b and h are length, width and thickness of the bar, respectively.

© 2020 by the authors. Submitted for possible open access publication under the terms and conditions of the Creative Commons Attribution (CC BY) license (http://creativecommons.org/licenses/by/4.0/).
